# Age-associated alterations in thalamocortical structural connectivity in youths with a psychosis-spectrum disorder

**DOI:** 10.1038/s41537-023-00411-7

**Published:** 2023-12-11

**Authors:** Lydia Lewis, Mary Corcoran, Kang Ik K. Cho, YooBin Kwak, Rebecca A Hayes, Bart Larsen, Maria Jalbrzikowski

**Affiliations:** 1 Institute of Child Development, University of Minnesota, Minneapolis, MN USA; 2 Department of Psychiatry and Behavioral Sciences, Boston Children’s Hospital, Boston, MA USA; 3 Psychiatry Neuroimaging Laboratory, Department of Psychiatry, Brigham and Women’s Hospital, Harvard Medical School, Boston, MA USA; 4 Department of Brain and Cognitive Sciences, College of Natural Sciences, Seoul National University, Seoul, Republic of Korea; 5 Department of Pediatrics, Masonic Institute for the Developing Brain, University of Minnesota, Minneapolis, MN USA; 6 Department of Psychiatry, Harvard Medical School, Boston, MA USA

**Keywords:** Psychosis, Biomarkers, Neural circuits

## Abstract

Psychotic symptoms typically emerge in adolescence. Age-associated thalamocortical connectivity differences in psychosis remain unclear. We analyzed diffusion-weighted imaging data from 1254 participants 8–23 years old (typically developing (TD):*N* = 626, psychosis-spectrum (PS): *N* = 329, other psychopathology (OP): *N* = 299) from the Philadelphia Neurodevelopmental Cohort. We modeled thalamocortical tracts using deterministic fiber tractography, extracted Q-Space Diffeomorphic Reconstruction (QSDR) and diffusion tensor imaging (DTI) measures, and then used generalized additive models to determine group and age-associated thalamocortical connectivity differences. Compared to other groups, PS exhibited thalamocortical reductions in QSDR global fractional anisotropy (GFA, *p*-values range = 3.0 × 10^–6^–0.05) and DTI fractional anisotropy (FA, *p*-values range = 4.2 × 10^–4^–0.03). Compared to TD, PS exhibited shallower thalamus-prefrontal age-associated increases in GFA and FA during mid-childhood, but steeper age-associated increases during adolescence. TD and OP exhibited decreases in thalamus-frontal mean and radial diffusivities during adolescence; PS did not. Altered developmental trajectories of thalamocortical connectivity may contribute to the disruptions observed in adults with psychosis.

## Introduction

Psychosis typically emerges in adolescence and early adulthood^1^, a period characterized by ongoing refinement of connections between the cortex and subcortical structures, including the thalamus^2–6^. The thalamus relays incoming sensory information to the cortices for further processing and is considered a hub for cognition and emotional processing^7^. Given that sensory, cognitive, and emotional processes are disrupted in psychotic disorders, the thalamus and its connected neural circuitry likely contribute to cognitive impairments and symptoms observed in psychosis^8,9^. In support of this view, researchers consistently find disruptions in structural and functional thalamocortical connectivity across the phases of psychotic illness^10–30^. However, the degree to which age-associated disruptions in thalamocortical connectivity contribute to psychosis remains unclear. Evaluating the age-associated patterns of thalamocortical structural connectivity in youth across the psychosis spectrum could help elucidate the neural basis of impairments associated with psychosis.

Studies of typical development find that white matter integrity in thalamocortical connections, as measured by fractional anisotropy (FA), increases with age^3,4,31,32^. In adults with schizophrenia, studies find reduced FA in white matter connectivity between the thalamus and prefrontal cortices;^21,22,33–35^ some also find lower FA in the thalamus-occipital and thalamus-parietal connections^33^. In a study that used probabilistic fiber tracking, in comparison to typically developing youth, psychosis-spectrum youth exhibited significantly lower FA values in tracts between the thalamus and six cortical regions (prefrontal, motor, somatosensory, temporal, posterior parietal and occipital cortices)^36^. This study found that typically developing and psychosis-spectrum youth exhibited linear age-related increases in FA between thalamus and motor and somatosensory cortices and linear age-related decreases in FA between thalamus and temporal and occipital cortices^36^.

These studies are foundational to our understanding of white matter alterations in psychosis, but we may learn more information by integrating recent developments in diffusion imaging methodology. First, most diffusion-weighted imaging studies of psychosis do not remove participants based on motion, though removing such poor quality scans influences age-associated patterns of neurodevelopment^37^. Second, prior DWI studies have used classical, model-based approaches to measure diffusion, which are limited in their ability to resolve water diffusion in voxels with multiple fiber directions. Model-free approaches, such as Q-Space Diffeomorphic Reconstruction (QSDR), improve upon the classical model-based approaches by more flexibly estimating water diffusion along multiple directions of diffusion within a voxel^38^. Specifically, in contrast to model-based approaches like diffusion tensor imaging which assume a known model of diffusion orientation, QSDR is a model-free approach that does not make assumptions about the distribution of water diffusion within a voxel. Instead, QSDR empirically estimates the orientation distribution function, which reflects the density of diffusion along multiple fiber directions. The model-free QSDR approach has advantages over traditional methods such as DTI in voxels that contain crossing fibers because it can independently estimate diffusion along each fiber direction, leading to improved accuracy in fiber tracking and enabling greater sensitivity to tissue alterations. The degree of diffusion along a particular fiber direction can then be measured as quantitative anisotropy (QA)^39,40^. Whereas tensor-based measures like FA measure the overall anisotropy within a voxel, QA is a measure of anisotropic diffusion along a particular fiber direction^41,42^. In a phantom study which used thin silica capillary tubes to simulate crossing fibers, QSDR tractography was shown to outperform DTI tractography^39^. In this same study, in vivo QSDR tractography in a human subject produced the least amount of false tracts when compared to DTI-aided or anatomy-aided tractography^39^.

Third, probabilistic tractography is the most common fiber tracking method used in psychosis studies^43–47^. An alternative approach is deterministic tractography, which defines a white matter fiber trajectory beginning in a seed region and proceeding along the primary diffusion direction for each subsequent voxel. This method, unlike probabilistic tractography, does not include randomization but relies on local fiber orientation; thus, the computed trajectory from a given seed will always be the same^48–50^. In an international tractography challenge where research groups performed tractography on a model with defined tracts based on the fiber bundles from the Human Connectome Project data set^51^, the deterministic QA tractography method was able to detect 92% valid connections while probabilistic tractography detected 45% of valid connections^52^. Thus, there is some evidence suggesting that QSDR is more sensitive to white matter microstructural organization than classical DTI. Researchers using the same dataset we utilized (i.e., Philadelphia Neurodevelopmental Cohort) published a study that used DTI and probabilistic tractography to examine group differences and age-effects in psychosis spectrum youth^36^. Using QSDR measures and deterministic tractography in this dataset may provide additional information regarding thalamocortical connectivity in psychosis-spectrum individuals. Finally, many developmental changes that occur during adolescence are nonlinear^53–55^ and these patterns may be obscured when analyzed using a linear model. Flexible modeling approaches like generalized additive models (GAMs), which do not require a linear relationship between the predictor and the development variable, should be considered.

Leveraging these methodological advances, we measured white matter diffusion using QSDR and used GAMs to (1) determine group differences and to (2) examine the extent to which age-associated thalamocortical connectivity differed in psychosis-spectrum youth. We then compare our findings to a classical model based DTI approach. We hypothesized that like earlier work^21,33–35^, DTI would capture group differences. Additionally, we hypothesized that when we used QSDR measures of thalamocortical connectivity, we would observe age-associated alterations in psychosis-spectrum youth that may be obscured in DTI measures^36^, and/or that the GAM models would capture distinct periods with specific age-associated alterations.

## Results

Below we report on group differences in QSDR and DTI measures (Study Goal 1). We then report the typical age-associated patterns observed in QSDR and DTI measures of thalamocortical tracts in TD, followed by discussion of statistically significant age-associated alterations observed in PS and/or OP (Study Goal 2). Group differences, age effects, and differences in age-related slopes between groups are reported in Supplementary Table 4. In the main text, we report on statistically significant group differences and periods of time when age-related smooths differed between TD and PS (i.e., ‘differences in smooths’). We also report when group and age-related differences were present in PS vs. OP. Discussion of descriptive statistics (i.e., time periods of age-related changes within a specific group) and periods of time when age-related slopes differed only between PS and OP are reported in the Supplementary Results.

### Psychosis-spectrum youth have reductions in GFA and FA thalamocortical connectivity

**Fig. 1:**
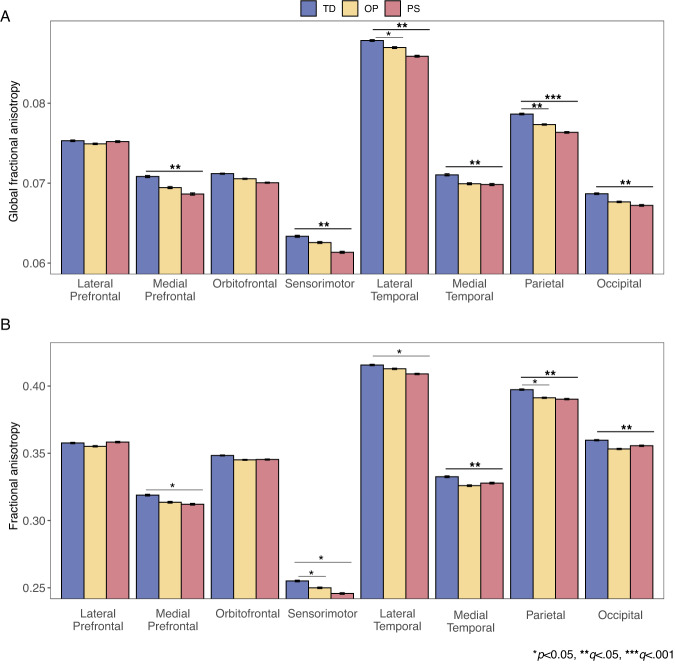
Group differences in two measures of thalamocortical connectivity. Group differences in thalamocortical fractional anisotropy (**A**) and global fractional anisotropy (**B**) in typically developing youth (blue) vs. youth with other psychopathologies (yellow) vs. psychosis-spectrum youth (red). The y-axis represents residualized data (age and sex regressed out) from the thalamocortical tracts, with the overall mean added to the residuals to facilitate one’s ability to interpret the findings.

In comparison to TD, PS exhibited reduced GFA in connections between the thalamus and the following cortices: medial prefrontal (*d* = 0.34), sensorimotor (*d* = 0.25), lateral temporal (*d* = 0.24), medial temporal (*d* = 0.24), parietal (*d* = 0.38), and occipital (*d* = 0.29, all *q* < 0.01, Supplementary Table 4, Fig. 1A, Supplementary Fig. 2A–H). OP had lower thalamus-parietal cortex GFA in comparison to TD (*d* = 0.26, *T* = −2.5,*p* = 0.01,*q* = 0.04), but not PS (*d* = 0.11, T = 1.8,*p* = 0.07,*q* = 0.17). OP did not exhibit disruptions in GFA when compared to either group (all *q* > 0.05) in any other thalamocortical tract.

Compared to TD, PS exhibited reductions in thalamus-medial temporal (*d* = 0.23), thalamus-parietal (*d* = 0.28), and thalamus-occipital FA (*d* = 0.29, all *q* < 0.0068, Supplementary Table 4, Fig. 1B, Supplementary Fig. 2I–P). OP did not differ from TD or PS on any FA measures (all *q* > 0.07). PS exhibited reduced thalamus-parietal AD in comparison to TD (*d* = 0.18, *q* = .05, Supplementary Table 4). OP did not exhibit significant differences in AD when compared to either group (all *q* > 0.07).

We re-ran all analyses co-varying for whole brain measures of FA and GFA. After correcting for multiple comparisons, only the thalamus-parietal GFA was significantly lower in PS vs. TD (*d* = 0.27, T = −2.6, *p* = 0.009, *q* = 0.02).

Main effects of sex are reported in the Supplementary Text and Supplementary Tables 5, 6.

### Psychosis-spectrum youth have age-associated alterations in prefrontal thalamocortical GFA and FA

**Fig. 2:**
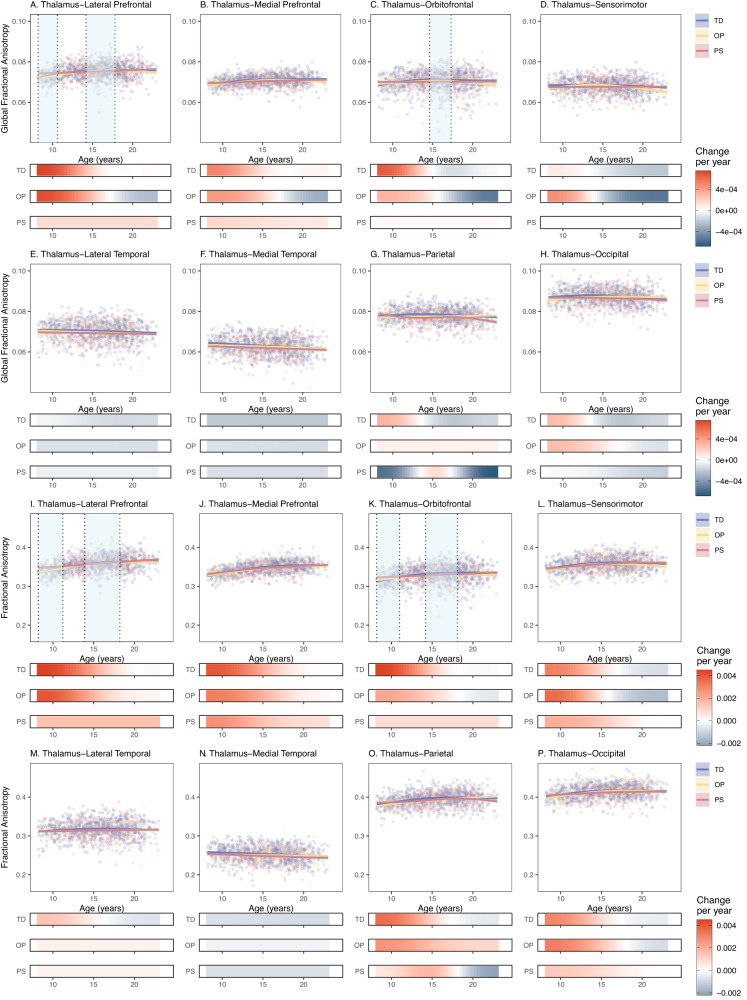
Neurodevelopmental trajectories of global fractional anisotropy and fractional anisotropy for psychosis-spectrum youth, typically developing youth, and youth with other psychopathologies. Partial residual plots of (**A**–**H)** global fractional anisotropy trajectories, and (**I**–**P)** fractional anisotropy trajectories, in tracts between the thalamus and the lateral prefrontal cortex (**A**, **I**), medial prefrontal cortex (**B**, **J**), orbitofrontal cortex (**C**, **K**), sensorimotor cortex (**D**, **L**), lateral temporal cortex (**E**, **M**), medial temporal cortex (**F**, **N**), parietal cortex (**G**, **O**), and occipital cortex (**H**, **P**) for typically developing youth (blue) youth with other psychopathologies (yellow) and psychosis-spectrum youth (red). The partial residual plots reflect the relationship between age (x-axis) and the respective neuroimaging measures (y-axis), given the covariates in the model. For each group, the thick line reflects the line of best fit. The bars underneath the age plots reflect the derivative of the slope, i.e., the rate of change taking place at a particular age. Darker blue indicates that there is a stronger decrease in the respective QSDR or DTI measure taking place at that age, while brighter red indicates a stronger increase in the respective QSDR or DTI measure. Dotted lines and areas of lightly shaded blue indicate times when there was a significant “difference in smooths” between typically developing and psychosis spectrum youth.

Both TD and OP exhibited age-associated increases in thalamus-lateral prefrontal GFA from 8.2 to 16 years. PS did not exhibit significant age-associated patterns in lateral-prefrontal GFA (Supplementary Table 4, Fig. 2A). Difference in smooths revealed that, from 8.2 to 10.6 years old, PS exhibited a shallower increasing slope in thalamus-lateral prefrontal GFA compared to both TD and OP (Supplementary Table 4, Fig. 2A). However, during the teenage years (14.2–17.8 years), difference in smooths revealed that TD age-associated patterns in thalamus-lateral prefrontal GFA plateaued when compared to PS, who exhibited a more linear age-related increase (Fig. 2A). Difference in smooths also revealed that OP exhibited an inverted u-shape change in thalamus-lateral prefrontal GFA during this same period when compared to PS (Fig. 2A). TD also exhibited a significant age-associated increase in thalamus-orbitofrontal GFA from 8.2 to 15.3 years (Supplementary Table 4, Fig. 2C). PS and OP did not exhibit an age-associated effect in this tract. Difference in smooths revealed that PS failed to exhibit the inverted u-shaped age-related slope that TD exhibited during adolescence (14.6–17.3 years) in thalamus-orbitofrontal GFA (Fig. 2C). Derivate plots in Fig. 2C show that, during this time, TD exhibited a significant increase and a subsequent decrease in thalamus-orbitofrontal GFA, but PS do not show any age-associated effects.

We observed a similar pattern of results for thalamus-prefrontal FA measures. TD and OP exhibited age-associated increases in thalamus-lateral prefrontal FA from 8.2 to 17 years, and PS showed an age-associated increase in thalamus-lateral prefrontal FA from 8.2 to 23 years (Supplementary Table 4, Fig. 2I). Difference in smooths revealed that, in comparison to TD, from 8 to 11 years old, PS showed a shallower age-related increase in thalamus-lateral prefrontal FA. However, during the teenage years (14–18 years), difference in smooths revealed that TD and OP thalamus-lateral prefrontal FA age-associated patterns plateaued when compared to PS, who exhibited a more linear age-related increase in thalamus-lateral prefrontal FA (Fig. 2I). TD and OP also exhibited significant age-associated increases in thalamus-orbitofrontal FA from 8.2 to 16 years (Supplementary Table 4, Fig. 2K). PS did not exhibit an age-associated effect in this tract. Difference in smooths revealed that, from 8.2 to 11 years old, PS showed a shallower age-related increase in thalamus-orbitofrontal FA when compared to TD (Fig. 2K). However, during the teenage years (14–18 years), TD thalamus-orbitofrontal FA trajectory plateaued when compared to PS, who exhibited a more linear age-related increase in thalamus-orbitofrontal FA during this period (Fig. 2K). PS did not show an altered age-related increase in thalamus-orbitofrontal FA in comparison to OP.

### Psychosis-spectrum youth exhibit age-related alterations in thalamus-frontal RD

TD exhibited age-associated decreases in thalamus-medial prefrontal and thalamus-orbitofrontal RD from middle childhood through adolescence. OP exhibited a similar pattern in thalamus-medial prefrontal RD, but PS did not show a significant effect of age in either tract. Difference in smooths revealed that, in comparison to TD, PS failed to show the u-shaped age-related effect (i.e., a significant decrease in RD followed by a significant increase in RD) during adolescence (14–17 years) in thalamus-medial prefrontal and thalamus-orbitofrontal RD (Supplementary Fig. 3B–C). For RD in the thalamus-sensorimotor tract, TD exhibited a u-shaped age effect, with significant *decreases* in RD from 8 to 14 years old, followed by significant age-associated *increases* in RD from 16 to 23 years (Supplementary Fig. 3D). PS and OP failed to exhibit any significant age effects. Differences in smooths revealed that from ~13 to 17 years old, PS failed to show the u-shaped age-associated effect exhibited by TD and OP (first a steeper decrease, followed by a steeper increase) in thalamus-sensorimotor RD (Supplementary Fig. 3D).

### Psychosis-spectrum youth do not exhibit typical age-related decreases in prefrontal MD

TD exhibited significant decreases in thalamus-medial prefrontal MD from 8 to 15 years, while PS and OP did not show this effect (Supplementary Fig. 4B). Difference in smooths revealed that PS failed to show the u-shaped age-associated effect that TD exhibited during adolescence (14.9–17.2 years) in thalamus-medial prefrontal MD (Supplementary Fig. 4B). Derivative plots in Supplementary Fig. 4B show that with increasing age during this period, TD exhibited significant decreases in MD, followed by significant increases, while PS did not. A similar pattern was observed between TD and PS in thalamus-sensorimotor MD between 13.7 and 16.9 years of age (Supplementary Fig. 4D).

PS and TD did not exhibit age-related differences in slopes in measures of QA, ISO, RDI, or AD.

## Discussion

Using a flexible statistical modeling technique (GAM) and deterministic tractography, we assessed group and age-related differences in thalamocortical structural connectivity in typically developing youth (TD), psychosis-spectrum youth (PS), and youth with other psychopathologies (OP). In comparison to TD and OP youth, PS youth exhibited widespread thalamocortical reductions in measures of QSDR-derived global fractional anisotropy (GFA) and DTI-derived fractional anisotropy (FA). Furthermore, in comparison to TD and OP, PS exhibited age-associated alterations in thalamus-lateral prefrontal and thalamus-orbitofrontal GFA and FA. Our results provide a novel view of developmental alterations in thalamocortical connectivity in youth experiencing psychosis spectrum symptoms which implicates differences in age-associated slopes at discrete periods of development in thalamus-prefrontal connections, as well as widespread group differences in thalamocortical connectivity metrics.

Previous studies of adults with psychosis have found widespread lower FA across white matter tracts^17,18,21–23,56^. Similarly, we found widespread reductions in thalamocortical tracts of PS youth with DTI FA and with a QSDR analogue, GFA. In a study that used the same PNC sample but used DTI FA and probabilistic tractography to map thalamocortical tracts, researchers found strikingly similar results with regards to DTI FA: PS youth exhibited lower FA levels across all tracts in comparison to TD youth^36^. Because this study^36^ and our study used the same data set, the agreement of the results is not surprising. However, we also examined thalamocortical connectivity to three separate prefrontal regions, allowing us to obtain more refined prefrontal cortical information. We found that group differences in PS were restricted to thalamus-medial prefrontal connections, but not thalamus-lateral prefrontal or -orbitofrontal tracts, suggesting that group differences are specific to that connection. Thalamocortical connectivity disruptions were present in early stages of psychotic illness and are observed in individuals as young as eight years old with sub-threshold and full-blown psychotic symptoms. Furthermore, these findings suggest that FA reductions reported in the Avery manuscript^36^ are not driven by issues commonly associated with the tensor model, such as crossing fibers.

We also found that, in comparison to TD and OP, PS exhibited age-associated alterations in FA and GFA thalamus-lateral prefrontal and thalamus-orbitofrontal tracts only (Fig. 2). Compared to TD and OP, PS youth exhibited a significantly shallower increase in thalamus-lateral prefrontal GFA and FA and in middle childhood (~8–10 years). A similar pattern of results was observed for thalamus-orbitofrontal structural connectivity in PS. In line with this work, reports of two cohorts of young people at elevated risk for developing psychosis found shallower age-associated slopes in measures of cortical thickness in the psychosis risk groups during middle to late childhood^57,58^. These findings may mean that neural circuitry is more strongly affected in youth who experience psychosis-spectrum symptoms at an earlier age. Alternatively, it is possible that PS youth had “precocious” decreases in connectivity that occurred prior to the age range we studied. In support of this hypothesis, there is some evidence that the brains of individuals with psychosis “age” faster^59^. However, these observations are speculative, and the veracity of these patterns will be most accurately captured with longitudinal analyses that encompass a wider age range.

During adolescence (ages 14–17 years), PS youth exhibited a more linear age-associated increase in thalamus-lateral prefrontal and thalamus-orbitofrontal tracts, when TD and OP youth exhibited a plateau. Adolescence has been conceptualized as a period of specialization when cellular mechanisms and neural circuitry motivate experience-seeking behaviors^60^ which in turn stimulate experience-dependent plasticity, strengthening neural synchrony and refining cortical networks^60^. If the thalamus-prefrontal GFA and FA trajectories of typically developing youth during adolescence represent a period of specialization (when higher-level systems that contribute to adult outcomes are formed^61,62^), an abnormal trajectory during middle childhood and adolescence, as exhibited by psychosis-spectrum youth, could reflect impairments in optimal specialization, particularly in prefrontal thalamocortical tracts. It has also been proposed that adolescence may be a “sensitive” or “critical” period for prefrontal development, where there is increased plasticity in connections between the prefrontal cortex and other structures (e.g., thalamus), making these connections more susceptible to internal and external influences^63–66^. Here, age-associated alterations in the thalamus-prefrontal tracts in PS, as well as other studies that show age-related alterations in prefrontal regions^67^ and prefrontal connections^68^, lends support to the notion that psychiatric disorders, particularly those with onset during adolescence, may arise in correlation with disruptions to prefrontal connections during this sensitive/critical period.

A longitudinal analysis of individuals at clinical-high risk for developing psychosis, found that the high-risk group who went on to develop a psychotic disorder also exhibited adolescent age-associated alterations in whole brain tissue FA, as well as altered age-associated patterns in extracellular free-water, which putatively indexes unrestricted extracellular water content^69^. Like the patterns we observed in middle childhood (~8–10 years), clinical high risk individuals who developed psychosis displayed slower rates of growth. However, in both measures assessed, during adolescence, tissue FA and free water were higher in high-risk participants who developed psychosis, not lower, like our study found. These differences are likely because a multi-shell diffusion acquisition was used in this study. Furthermore, this study also used different diffusion indices. However, this study^69^ and our findings show that elevated risk for psychosis, as well as individuals with a psychotic disorder diagnosis, show subtle, age-related white matter alterations. In the future, it will be important to examine how free-water measures of diffusion, as well as tissue-specific FA measures, can complement QSDR and DTI white matter measures.

Age-related declines in white matter FA have also been observed in those with an established psychotic disorder^70,71^ and this is often interpreted, in combination with postmortem findings, as the breakdown of myelin. In our study, compared to TD, PS youth also exhibited age-associated abnormalities in thalamus-prefrontal RD, the DTI measure putatively associated with myelination^72–74^, when compared to typically developing youth. TD youth exhibited a u-shaped age-associated trajectory in RD from 14 to 17 years that was not exhibited by PS youth, which may indicate that myelination processes are altered in psychosis-spectrum youth during adolescence. In contrast, we did not find any age-associated abnormalities in AD, a DTI measure possibly associated with axonal density^73,75,76^. We did find age-associated differences in MD, the DTI measure that may be associated with the amount of water content^77,78^, when comparing PS youth to TD youth. However, caution is warranted regarding these neurobiological interpretations, given that the presence of multiple fiber populations and/or crossing fibers in white matter tracts complicate such explanations^79,80^.

Notably, other than the thalamocortical GFA alteration, we did not find other significant group differences or age-associated abnormalities with other QSDR measures when comparing PS to TD. Traditionally, QSDR measures are considered more sensitive and specific to white matter microstructural organization than DTI measures, as QSDR allows for multiple directions of diffusion within a voxel^38^. However, it is possible that multi-shell acquisition, diffusion spectrum acquisition, additional diffusion directions, and/or higher-resolution imaging is needed to detect group and age-associated differences in other QSDR measures^38,41^.

There are limitations to the present study. We analyzed a cross-sectional data set; therefore, the age-associated abnormalities identified in this study do not reflect within individual change. In the future, longitudinal studies of psychosis-spectrum youth may better identify how the shape and rate of maturation of subject-specific developmental trajectories in psychosis-spectrum youth differ in comparison to typically developing youth^32,81,82^. Moreover, because psychotic symptoms are often dynamic and change over time^83,84^, these changes need to be considered when characterizing neurodevelopmental change. Time-varying analytic approaches^85,86^ could be applied to longitudinal neuroimaging and psychotic symptom data to link psychotic symptoms to specific DWI measures at precise periods of development. Furthermore, although DWI can detect differences in structural connectivity, microstructural differences that result from neurobiological differences in myelination, axonal density, or water content may be better detected with higher-resolution imaging and/or comparisons with myelin-based imaging^87^ or free-water imaging^88^. We also failed to find significant differences between TD and OP. This lack of an effect may be due to the large heterogeneity of psychiatric diagnoses observed in this group (Supplementary Table 1). Systematic evaluation of thalamocortical tracts in other psychopathologies will be necessary to determine if these tracts are affected in youth with other psychiatric disorders, as thalamocortical connectivity is disrupted in adults with other psychiatric disorders^89^. Finally, in our analyses, we only examined the whole thalamus as a region of interest (ROI). Previous studies have found disrupted connectivity in the anterior and mediodorsal areas of the thalamus in individuals with schizophrenia^90^ and in the anterior, mediodorsal and pulvinar areas of the thalamus in first-episode individuals^91^. Altered connectivity in thalamic nuclei have been associated with cognitive deficits observed in psychosis-spectrum individuals, including attention (anterior^92^, pulvinar^93–96^ and mediodorsal^91,97,98^ nuclei) and working memory (anterior^92^ and mediodorsal^91,97,98^ nuclei). Future investigations with higher MRI scan resolution can look at thalamocortical development in terms of individual thalamic nuclei to understand whether a particular sub-nucleus is driving the group- and age-differences seen in our study.

Taken together, our results provide compelling evidence for age-associated disruptions of frontal white matter thalamocortical connectivity in psychosis-spectrum youth. In the future, we plan to test the extent to which these findings are observed in longitudinal data sets of youth over the course of development (e.g., the Adolescent Brain and Cognitive Development Study) to better understand how within-subject neurodevelopment contributes to the neurobiological mechanism that underlie psychosis onset.

## Methods

### Pre-registration of investigation

We completed pre-registration of this project using the Open Science Framework website. The project was titled “Age-associated alterations in thalamocortical structural connectivity in psychosis spectrum disorder” and was approved and registered in January 2019 (https://osf.io/es3cf). See the Supplementary Text for further details.

### Participants

The final neuroimaging dataset consisted of 1254 participants ages 8–23 years old from the Philadelphia Neurodevelopmental Cohort (PNC, Table 1). Inclusion criteria for PNC subjects included the ability to provide signed informed consent, English proficiency, and the ability to engage in psychiatric and cognitive phenotyping procedures. The subjects were recruited from a database at the Center for Applied Genomics at the Children’s Hospital of Philadelphia, as 78% of the 50,000 youths had provided consent to be re-contacted for future research. Exclusion criteria for the subset of subjects in the PNC who received neuroimaging included: medical problems that could impact brain function (such as severe medical problems, neurological conditions, or endocrine disorders), impaired vision/hearing, claustrophobia, or MRI contraindications.

**Table 1:** Participant baseline demographics.

	Typically Developing	Other Psychopathology	Psychosis- Spectrum	Significant Difference?	Direction of Effect
Total N	626	299	329	NA	NA
#F | %F	324 | 51.76%	176 | 58.86%	176 | 53.5%	*p* = 0.13	NA
Mean Age in years (SD)	15.38 (3.66)	14.91 (3.69)	15.89 (2.69)	*p* = 0.002	PS > OP & TD
Mean Total Negative Symptoms (SD)	1.30 (1.74)	1.51 (1.85)	5.03 (4.59)	*p* < 2.2e^-16^	PS > OP & TD
Mean Total Positive Symptoms (SD)	3.27 (1.74)	3.79 (1.85)	22.20 (4.59)	*p* < 2.2e^-16^	PS > OP & TD

*F* female. Total Negative symptoms were calculated from severity scores to six negative symptom items from the Scale of Prodromal Symptoms (i.e., attention and focus, disorganized speech, perception of self, experience of emotion, occupational function and avolition). Scores could range from 0 to 36, as an individual could receive a score from 0 to 6 for each negative symptom item. Total positive symptoms were calculated from 12-item self-report, the Prevention through Risk Identification, Management & Education Screen Revised^101^ (PRIME). For each item, participants could choose a value from 0 (completely disagree) to 6 (completely agree). Scores for the PRIME could range from 0 to 72.

We used responses from a modified version of the Kiddie-Schedule for Affective Disorders and Schizophrenia (KSADS) interview^99^, GOASSESS, to determine psychopathology history. The GOASSESS is a computerized tool for evaluating psychopathology domains^100^. Following completion of the GOASSESS, we defined psychosis-spectrum youth as participants who: (1) had a score of 6 on any Prevention through Risk Identification, Management & Education Screen Revised^101^ (PRIME) item; had a score of 5 or 6 on three or more items on the PRIME Screen Revised; (2) answered ‘yes’ to hallucination related questions on the KSADS, reported that they were not using drugs at the time the symptom was experienced, and endorsed experiencing significant impairment or distress as a result; or (3) scored 2 standard deviations or more above the age-cohort mean total score on six of the Scale of Prodromal Symptoms^102^ (SOPS) negative symptom items: attention and focus, disorganized speech, perception of self, experience of emotion, occupational function and avolition. Individuals in this psychosis spectrum group either fell into a group of individuals experiencing subthreshold level psychotic symptoms (*N* = 222, 67%) or met criteria for a psychotic disorder (*N* = 107, 33%), based on combined responses to the GOASSESS, SOPS, and PRIME. We defined typically developing youth as youth who denied clinically significant symptoms of psychopathology based on responses to the GOASSESS interview. To define the “other psychopathology” group, we used responses to questions on the GOASSESS to determine DSM-IV diagnosis ranking. Like other PNC publications^103,104^, we considered psychopathology to be significant if symptoms endorsed were consistent with frequency and duration of a DSM-IV psychiatric disorder, while correspondingly accompanied by significant distress or impairment (rating of >5 on a scale of 0–10). See Supplementary Table 1 for a breakdown of distinct DSM-IV psychiatric diagnoses observed in the other psychopathology group.

### MRI acquisition

All PNC scans were collected with a 32-channel head coil on a single 3 T Siemens Tim Trio whole-body scanner at the Hospital of the University of Pennsylvania. Diffusion-weighted imaging was split into two separate imaging runs, with a full scanning time of approximately 11 min. Scans were acquired with the following parameters: TR = 8100 ms, TE = 82 ms, FOV = 240 by 240 mm; Matrix = 128 × 128 x 70, in-plane resolution = 1.875 mm^2^; slice thickness=2 mm, gap = 0 mm; FlipAngle=90°/180°/180°, volumes = 71 (35 in first run, 36 in second run), GRAPPA factor = 3, bandwidth = 2170 Hz/pixel, PE direction = AP. The DWI sequence was a twice-refocused spin-echo (TRSE) single-shot EPI sequence, consisted of 64 b = 1000 s/mm^2^ diffusion-weighted volumes and 7 b = 0 s/mm^2^ volumes. In order to minimize eddy current artifacts^105^, a four-lobed diffusion encoding gradient scheme and a 90-180-180 spin echo sequence were used. For more details see Satterthwaite et al., 2014^106^.

### MRI processing

Consistent with previous studies^36,37^, we concatenated two separate runs for each subject into a single data set (71 volumes)^37^. We performed a Gibbs ringing artifact removal^107^ and then Eddy current-induced distortion and motion correction using Eddy, from FSL, with outlier removal function^108,109^. We then used the QSDR method, available in the DSI Studio Software^110^, to reconstruct the images and warp them to standard MNI space.

### Thalamic region of interest

**Fig. 3:**
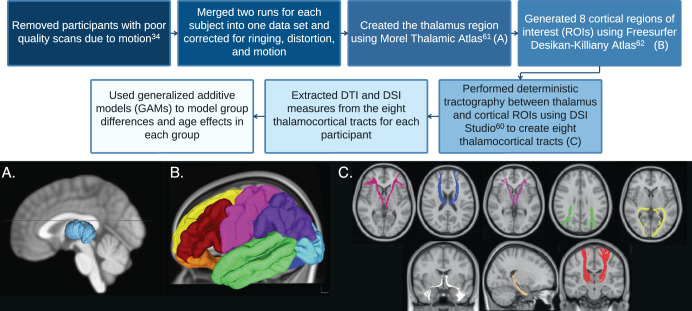
Diffusion weighted imaging analysis pipeline. **A** Thalamic region of interest (light blue). **B** Cortical regions of interest: the lateral prefrontal cortex (red), medial prefrontal cortex (yellow), orbitofrontal cortex (orange), sensorimotor cortex (pink), lateral temporal cortex (green), medial temporal cortex (dark blue), parietal cortex (purple), occipital cortex (light blue). **C** Tracts between the thalamus and the lateral prefrontal cortex (pink), medial prefrontal cortex (dark blue), orbitofrontal cortex (purple), sensorimotor cortex (red), lateral temporal cortex (white), medial temporal cortex (beige), parietal cortex (green), occipital cortex (yellow).

We created the thalamic ROI using the Morel Thalamic Atlas that was adapted to 3D MNI space^111^. We merged the individual thalamic nuclei to create the whole thalamus (i.e., merged anterior, mediodorsal, lateral geniculate, medial geniculate, ventral anterior, ventral lateral, ventral posterior lateral, ventral posterior medial, pulvinar, lateral dorsal, lateral posterior, and intralaminar regions). See Fig. 3A for a visual depiction. We restricted our analyses to the whole thalamus because we were not confident we could accurately measure thalamic nuclei with the DTI sequence resolution used in this study, as recent thalamic nuclei parcellations were created from scans with higher resolution^112,113^.

### Cortical parcellation

Similar to previous publications on thalamocortical connectivity^17,22^, we generated eight cortical ROIs (lateral prefrontal cortex, medial prefrontal cortex, orbitofrontal cortex, sensorimotor cortex, lateral temporal cortex, medial temporal cortex, parietal cortex, and occipital cortex) using the FreeSurfer Desikan-Killiany atlas^114^ as reported in Supplementary Table 2 and shown in Fig. 3B. We selected the above-mentioned cortical segmentation, with three different prefrontal regions, because it allowed us to study connectivity to the prefrontal cortex with a greater degree of specificity. Because we did not have any a priori hypotheses about laterality, we calculated bilateral connections between the thalamus and all cortical regions.

### Tractography

We performed deterministic tractography using DSI Studio^110^. We performed the first set of tracking between the bilateral whole thalamus and each bilateral cortical ROI using the Human Connectome Project group average template image of 1021 subjects^115^. We designated both the thalamus and the selected cortical region as ROIs and each of the seven other cortical regions as regions of avoidance. After performing tractography, we manually edited tracts to remove any fibers that strongly deviated. As shown in Supplementary Table 3, we conducted minimal manual editing and removed fibers only if they were clearly spurious (e.g., a fiber was “alone” or going in the opposite direction compared to the remaining bundle of fibers). The eight thalamocortical tracts are shown in Supplementary Fig. 1.

We performed tracking with a threshold QA of 0.1, an angular threshold of 60 degrees, and a 0.5 mm step size (half the size of one voxel). We used 30 mm as the minimum length and 120 mm as the maximum length and removed any tracts falling outside of these values. We terminated tracking after 5,000,000 seeds. We selected tractography parameters similar to previous publications that used deterministic QA-based tractography in DSI Studio^116–119^. After tractography, we converted these eight thalamocortical tracts into ROIs and extracted QSDR values from each subject for each of the eight tracts.

### Diffusion measures

Extracted DTI and QSDR measures from the eight thalamocortical tracts are described below.

### DTI measures

DTI measures assess the diffusion signal by using a tensor model^120,121^. FA estimates the anisotropy of diffusion of water molecules across a tissue and ranges between 0 (completely isotropic) and 1 (completely anisotropic). Axial diffusivity (AD) indicates the rate at which molecules diffuse in the primary diffusion direction, radial diffusivity (RD) measures the rate at which water molecules diffuse along the secondary and tertiary directions, and mean diffusivity (MD) measures the average amount of diffusion from all three axes. These measures have been associated with a wide range of neurobiological processes (e.g., FA is associated with white matter integrity^122^, axonal density has been linked to AD^73,75,76^), though multiple works demonstrate that we cannot draw strong biophysical interpretations from these measures, particularly when there are multiple fiber populations present and there are crossing fibers/ complex geometries associated with tracts^79,80^.

### QSDR measures

QSDR improves upon the classical diffusion tensor model by allowing for the possibility of multiple directions of diffusion within a single voxel^38^. QSDR measures are derived from a model-free approach that uses information from a diffusion orientation distribution function; evidence suggests these measures better represent complex fiber organization^39,123^. GFA is considered the QSDR analogue to FA. GFA ranges between 0 and 1 and corresponds to the magnitude of the principal diffusion direction in each voxel^123^. QA is a measure of anisotropic diffusion that incorporates spin density information^39^. Isotropy (ISO) measures background isotropic diffusion. Restricted diffusion imaging (RDI) indicates the total amount of restricted diffusion in a tissue in any orientation. Like DTI measures, there is evidence that QSDR measures are potential indicators of neurobiological processes, such as axonal density (QA^124,125^), cerebrospinal fluid and edema (ISO^124^), and cell density and inflammation (RDI^126^).

### Image quality assessment

To assess image quality and remove participants with poor quality scans due to motion, we calculated a temporal signal-to-noise ratio (TSNR), based on a previous publication^37^. We estimated TSNR at each brain voxel for the 64 b = 1000 s/mm^2^ DTI volumes. We then obtained a single SNR measure by averaging all brain voxel TSNRs. We chose this measure to assess image quality because it differentiated poor data from usable data with a high degree of accuracy^37^. We used the defined cut-off point of 6.47 to exclude poor quality scans from our analyses^37^.

### Statistics

All analyses were conducted using R version 4.2.2^127^. We used the R package mgcv^128^ to conduct general additive models (GAMs) to examine group differences (typically developing vs. psychosis spectrum vs. other psychopathology) and smoothed age effects in each group on all diffusion measures. A GAM is an extension of the general linear model but does not assume a linear relationship between the predictor and dependent variable, allowing for a more flexible relationship. To avoid overfitting, GAMs assess a penalty on nonlinearity. Smoothed predictor functions can flexibly capture linear and nonlinear effects with a set of basis functions. In this study, age is modeled as a smooth function, which is represented using penalized regression splines, setting an upper limit of k = 3 basis dimensions. Fitting was performed using restricted maximum likelihood. We utilized a Gaussian distribution and assessed model fit by examining residual plots and diagnostic tests that assessed the appropriateness of chosen basis dimensions. Because the relationship between the smoothed predictor and the dependent variable is not required to have the same functional form in each group, we were able to examine the smoothed effects of chronological age for all groups separately. We included sex in all models as a covariate. After running all models, we used False Discovery Rate to correct for multiple comparisons of group effects *N* = 192, three comparisons [TD vs. OP; TD vs. PS; PS vs. OP] for each of the eight DTI/QSDR measures [FA, AD, RD, MD, GFA, QA, ISO, RDI] for eight thalamocortical tracts [thalamus-lateral prefrontal, thalamus-medial prefrontal, thalamus orbitofrontal, thalamus-sensorimotor, thalamus-lateral temporal, thalamus-medial temporal, thalamus-parietal, and thalamus-occipital] and age effects (*N* = 192, age effects for three separate groups for eight DTI/QSDR measures for eight tracts) and obtain a corrected p-value (i.e., *q*-value).We used the R package emmeans^129^ to conduct post-hoc analyses of statistically significant effects of group and sex.

To determine time periods in which significant change was occurring in each group, we used the R package gratia^130^ to estimate a multivariate normal distribution whose vector of means and covariance were defined by the fitted GAM parameters to simulate 10,000 GAM fits and their first derivatives, generated at 0.1-year age intervals. Similar to previous publications^57,61,131^ and in line with recent guidelines^132^, we defined significant intervals of age-related change in MRI measures as ages when the 95% confidence intervals of simulated GAM fits did not include zero.

To determine time periods during which age effects in each group differed from another group (e.g., typically developing age effects vs. psychosis spectrum age effects), we also used the R package gratia^130^ to take the difference between the upper and lower 95% confidence intervals of the smoothed fit in two groups, henceforth called the ‘difference in smooths’. For each dependent variable, we considered effects of age to be significantly different in the two groups being compared during periods of time in which the difference in smooths did not include zero. This approach has been used in previous publications^57,133,134^.

### Supplementary information

Supplemental Material

## Data Availability

All Philadelphia Neurodevelopmental Cohort (PNC) data used in this study is publicly available through the database of Genotypes and Phenotypes (***dbGaP***). The PNC is listed under the original project name: “Neurodevelopmental Genomics: Trajectories of Complex Phenotypes. ” The full URL is https://www.ncbi.nlm.nih.gov/projects/gap/cgi-bin/study.cgi?study_id=phs000607.v3.p2 All code used to merge, clean, and analyze data used in this study can be found here: https://github.com/bchnerdlab/PNC_thalamocortical_dwi
