# Supplementary material for: Age-associated alterations in thalamocortical structural connectivity in youths with a psychosis-spectrum disorder
Source: Schizophrenia (Heidelb). 2023 Dec 11;9(1):86. doi: 10.1038/s41537-023-00411-7 (PMC10713597; doi:10.1038/s41537-023-00411-7)

**Supplementary Materials for “Age-associated alterations in thalamocortical structural connectivity in youths with a psychosis-spectrum disorder”**

Table of Contents

[Supplementary Methods 3](#_Toc146724382)

[*Project Timeline of Study Pre-Registration* 3](#_Toc146724383)

[Supplementary Results 3](#_Toc146724384)

[*Post-hoc Analyses of Sex Effects* 3](#_Toc146724385)

[*Additional Age Associated Effects in Quantitative Anisotropy (QA)* 4](#_Toc146724386)

[*Additional Age Associated Effects in Global Fractional Anisotropy (GFA)* 4](#_Toc146724387)

[*Additional Age Associated Effects in Isotropic Diffusion (ISO)*  4](#_Toc146724388)

[*Additional Age Associated Effects in Restricted Diffusion imaging (RDI)* 4](#_Toc146724389)

[*Additional Age-Associated Effects in Fractional Anistropy (FA)*  5](#_Toc146724390)

[*Additional Age-Associated Effects in Mean Diffusivity (MD)_* 5](#_Toc146724391)

[*Additional Age-Associated Effects in Radial Diffusivity (RD)_* 5](#_Toc146724392)

[*Additional Age-Associated Effects in Axial Diffusivity* 6](#_Toc146724393)

[Supplementary Table 1 7](#_Toc146724394)

[Supplementary Table 2. 8](#_Toc146724395)

[Supplementary Table 3. 9](#_Toc146724396)

[Supplementary Table 4. 11](#_Toc146724397)

[Supplementary Table 5. 16](#_Toc146724398)

[Supplementary Table 6.DTI effects of sex for thalamocortical tracts. Bold text denotes significant effects. 17](#_Toc146724399)

[Supplementary Figure 1. 20](#_Toc146724400)

[Supplementary Figure 2 21](#_Toc146724401)

[Supplementary Figure 3 22](#_Toc146724402)

[Supplementary Figure 4 23](#_Toc146724403)

# **Supplementary Methods**

## *Project Timeline of Study Pre-Registration*

This project initially began as Lydia Lewis’ thesis for her undergraduate Neuroscience degree. Dr. Maria Jalbrzikowski served as thesis advisor. The thesis project, *Age-associated alterations in thalamocortical structural connectivity in first episode psychosis,* was registered on the Open Science Framework (OSF) website, in October of 2018 (<https://osf.io/dy36r>). The thesis project was completed in December 2018 and initial results were intriguing but did not survive statistical corrections. To ensure adequate power in our analyses, we decided to work with a larger data set, the publicly available Philadelphia Neurodevelopmental Cohort. We pre-registered the expanded follow-up project in January of 2019 (<https://osf.io/es3cf>) and began analysis in the new cohort. In addition, we slightly altered our hypotheses due to the results obtained from the original project.

# **Supplementary Results**

The supplementary results describe the main effects of sex as well as additional GAM statistics we obtained in the age-associated analyses. Specifically, we report tracts in which QSDR and/or DTI values significantly differed between males and females. Additionally, we focus on time periods of statistically significant age-related changes within a group (i.e. psychosis spectrum (PS), typically developing (TD), other psychopathology (OP)) and periods of time when age-related slopes differed only between psychosis-spectrum youth. We report findings from QSDR and DTI measures.

## *Post-hoc Analyses of Sex Effects*

In comparison to females, males exhibited higher FA values in connections between the thalamus and the following cortices: lateral prefrontal (d=0.2), medial prefrontal (d=0.3), orbitofrontal (d=0.2), sensorimotor (d=0.2), lateral temporal (d=0.2), and parietal (d=0.2, Supplementary Table 5). Females had higher MD values than males in tracts between the thalamus and lateral prefrontal (d=0.1), medial prefrontal (d=0.1), orbitofrontal (d=0.2), and parietal cortices (d=0.2, Supplementary Table 5). Males also had lower RD values compared to females in tracts between the thalamus and medial prefrontal (d=0.1), orbitofrontal (d=0.2), and parietal (d=0.2) cortices and lower AD values compared to females in the thalamus-orbitofrontal tract (d=0.1, Supplementary Table 5).

Compared to females, males exhibited higher GFA values in connections between the thalamus and the medial prefrontal (d=0.2), sensorimotor (d=0.2), lateral temporal (d=0.2), parietal (d=0.2), and occipital cortices (d=0.2, Supplementary Table 6). Females had higher QA values than males in multiple thalamocortical tracts: lateral prefrontal (d=0.2), medial prefrontal (d=0.3), orbitofrontal (d=0.1), sensorimotor (d=0.2), and medial temporal (d=0.2, Supplementary Table 6). Males had a higher RDI value than females in the thalamus-occipital tract (d=0.1), but females had higher RDI values than males in all other thalamocortical tracts (Supplementary Table 6). Males had a higher thalamus-occipital ISO value than females (d=0.1), but females had higher thalamus-lateral prefrontal (d=0.2), -medial prefrontal (d=0.3), -orbitofrontal (d=0.2), -sensorimotor (d=0.2), -lateral temporal (d=0.1), and -medial temporal (d=0.2) ISO values than males (Supplementary Table 6).

## *Additional Age Associated Effects in Quantitative Anisotropy (QA)*

For thalamus-lateral prefrontal and orbitofrontal QA, typically developing youth exhibited an inverted u-shaped trajectory, with significant *increases* in QA from 8.2~12 years old, followed by age-associated decreases in QA from 16-23 years. PS and OP failed to exhibit any thalamus-prefrontal QA age-associated effects. For all groups, QA of the thalamus-lateral temporal, thalamus-medial temporal, and thalamus-occipital tracts showed significant age-associated decreases from middle childhood (8.2 years of age) through early adulthood (23 years of age). In the thalamus-parietal tract, TD and OP exhibited a similar linear decrease in QA between 8.2 and 23 years of age, while PS exhibited a linear decrease in this tract between 12.4 and 23 years of age. All groups exhibited age-associated decreases in QA in the white matter tract connecting the thalamus to the sensorimotor cortex beginning in their early teens (~14 years of age) through young adulthood (23 years of age).

## *Additional Age Associated Effects in Global Fractional Anisotropy (GFA)*

Both TD and OP exhibited age-associated increases in GFA from 8.2~16 years of age in thalamus-prefrontal tracts, while PS failed to show any age-associated effects in these connections. TD also exhibited a similar age-associated increase (8.2-15.3 years) in thalamus-orbitofrontal GFA, while OP and PS youth did not show this pattern. Between 8.2-23 years, TD exhibited a significant age-associated decrease in thalamus-medial temporal GFA; OP and PS both failed to exhibit these effects. OP exhibited an inverted u-shaped trajectory in thalamus-sensorimotor GFA, with a significant age-associated increase in GFA from 8.2-12.4 years, followed by a significant age-associated decrease in GFA from 15.2-23 years. PS and TD failed to exhibit age-associated effects in GFA of the thalamus-sensorimotor tract.

## *Additional Age Associated Effects in Isotropic Diffusion (ISO)*

In the thalamus-lateral prefrontal, thalamus-medial prefrontal, and thalamus-orbitofrontal tracts, TD youth exhibited significant age-associated decreases in isotropy between ages ~14-23 years. In OP youth, the age range was extended for thalamus-lateral prefrontal and thalamus-orbitofrontal ISO decreases (8.2-23). PS youth failed to exhibit age-associated changes in ISO of thalamus-lateral prefrontal, thalamus-medial prefrontal, or thalamus-orbitofrontal tracts. All groups exhibited significant age-associated linear decreases in ISO in the remaining thalamocortical tracts (thalamus-lateral temporal, thalamus-medial temporal, thalamus-parietal, thalamus-occipital, thalamus-sensorimotor) between ~9 and 23 years of age.

*Additional Age Associated Effects in Restricted Diffusion imaging (RDI)*

For thalamus-lateral prefrontal, thalamus-medial prefrontal, and thalamus-orbitofrontal RDI, TD exhibited age-associated decreases from ~14-23 years of age. PS failed to exhibit age-associated effects in RDI of all three observed thalamus-prefrontal tracts, while OP failed to exhibit age-associated affects in RDI of thalamus-lateral prefrontal and thalamus-medial prefrontal tracts. OP showed age-associated decreases in the thalamus-orbitofrontal RDI between 8.2-23 years of age. All groups exhibited linear decreases in RDI for connections between the thalamus and lateral temporal, medial temporal, parietal, occipital, sensorimotor cortices from childhood to early adulthood (~9~23 years).

## *Additional Age-Associated Effects in Fractional Anistropy (FA)*

TD and OP exhibited increases in FA in three of the observed thalamocortical tracts (thalamus-orbitofrontal, thalamus-parietal, and thalamus-occipital) from 8.2~17 years of age. All groups exhibited significant increases in FA starting at 8.2 years in FA of the thalamus-lateral prefrontal, thalamus-medial prefrontal, and thalamus-sensorimotor tracts. In the thalamus-lateral prefrontal tract, the increase in FA occurred between 8.2~17 years of age in TD and OP, whereas it occurred from 8.2-23 years of age in PS. For all three groups, FA in the thalamus-medial prefrontal tract increased between 8.2~18 years of age. All three groups exhibited significant increases in FA in connections between the thalamus and sesnorimotor cortex between 8.2~16 years of age. However, for TD and OP, the increase in FA was followed by a significant decrease in FA in early adulthood (21-23 years of age); this age-associated effect was not observed for PS. TD exhibited a significant increase in FA in the thalamus-medial temporal tract between 10.5-23 years of age; this effect was not observed for OP and PS.

## *Additional Age-Associated Effects in Mean Diffusivity (MD)_*

Typically developing youth and OP exhibited significant decreases in MD from 8.2~16 years of age in the thalamus-parietal tract; however, PS youth failed to exhibit similar age-associated changes. Between 8.2~15 years of age, TD also exhibited age-associated decreases in MD in thalamus-medial prefrontal, thalamus-orbitofrontal and thalamus-sensorimotor tracts. The decrease in thalamus-sensorimotor MD observed in TD was followed by a significant increase in MD between 17.5-23 years of age. These age-associated effects were absent in OP and PS. Notably, youth with other psychopathology exhibited a u-shaped trajectory in thalamus-occipital MD, with significant *decreases* in MD from 8.2~14.4 years of age, followed by an age-associated increase in MD from 17-23 years of age.

## *Additional Age-Associated Effects in Radial Diffusivity (RD)_*

Typically developing youth exhibited age-associated decreases in RD from middle childhood through adolescence (8.2~16 years of age) in six of the eight thalamocortical tracts (thalamus-lateral prefrontal, thalamus-medial prefrontal, thalamus-orbitofrontal tracts. OP exhibited a similar trend in thalamus-medial prefrontal RD, while PS failed to exhibit any age associated changes in thalamus-frontal RD. TD exhibited a u-shaped trajectory in thalamus-sensorimotor and thalamus parietal RD, with significant decreases from 8.2~15 years of age, and significant increases from ~17-23 years of age. OP exhibited a significant increase in thalamus-parietal RD between 8.2-15.7 years of age, while PS failed to exhibit significant age effects in the thalamus-sensorimotor and thalamus-parietal tracts. OP exhibited a u-shaped trajectory in thalamus-occipital RD, with a significant decrease in RD between 8.2-14.5 years of age and a significant between 17.1-23 years of age. TD exhibited a significant linear increase in thalamus-medial temporal RD from 8.2-23 years of age; OP and PS both failed to exhibit this age effect.

## *Additional Age-Associated Effects in Axial Diffusivity*

Typically developing youth exhibited age-associated decreases in AD in three of the eight thalamocortical tracts: thalamus-sensorimotor, thalamus-occipital, and thalamus-parietal; OP exhibited an age-associated decrease in AD in only the thalamus-parietal tract, while PS did not exhibit any age-associated changes in AD. For TD, age-associated changes occurred from 8.2~16 years of age in the thalamus-sensorimotor and thalamus-parietal tracts. From 8.2-23 years of age, linear decreases in AD occurred for TD in the thalamus-occipital tract and occurred for OP in the thalamus-parietal tract.

**Supplementary Table 1***.* Type and number of psychiatric diagnoses that were calculated from individuals in the “Other Psychopathology” group. Diagnoses are not mutually exclusive.

| **DSM-IV Diagnosis** | **N (%) that meet criteria for diagnosis** |
| --- | --- |
| Major Depressive Disorder | 18 (5%) |
| Generalized Anxiety Disorder | 3 (0.8%) |
| Obsessive Compulsive Disorder | 44 (11%) |
| Panic Disorder | 0 (0%) |
| Agoraphobia | 14 (4%) |
| Specific Phobia | 244 (65%) |
| Post-traumatic Stress Disorder | 53 (14%) |
| Social Anxiety Disorder | 21 (6%) |
| Anorexia | 6 (1.6%) |
| Other Eating Disorder (Binge Eating Disorder, Bulimia) | 6 (1.6%) |
| Autism Spectrum Disorder | 9 (2%) |
| Attention-deficit/hyperactivity Disorder | 17 (4.4%) |
| Conduct Disorder | 11 (3%) |
| Oppositional Defiant Disorder | 61 (16%) |

# **Supplementary Table 2.**

Cortical regions of interest (ROI) used as tractography targets in this investigation. Next to each cortical ROI is a list of the regions in the Freesurfer Desikan-Killiany atlas that were merged to create each ROI. The insula was not included in our study because initial tractography did not identify tracts between the thalamus and insula. A three-dimensional visualization of these ROIs is presented in Figure 1.

| **ROI** | **FreeSurferDKT regions included:** |
| --- | --- |
| Orbitofrontal Cortex (OFC) | Pars orbitalis, medial orbitofrontal cortex, lateral orbitofrontal cortex |
| Medial Prefrontal Cortex (MPFC) | Caudal anterior cingulate, rostral anterior cingulate, superior frontal gyrus |
| Lateral Prefrontal Cortex (LPFC) | Parts triangularis, rostral middle frontal gyrus, pars opercularis, caudal middle frontal gyrus |
| Sensorimotor Cortex (SMC) | Precentral gyrus, postcentral gyrus, paracentral lobule |
| Parietal Cortex (PC) | Inferior parietal cortex, supramarginal gyrus, precuneus cortex, posterior cingulate cortex, isthmus cingulate, superior parietal cortex |
| Medial Temporal Cortex (MTC) | Entorhinal cortex, parahippocampal gyrus, fusiform gyrus |
| Lateral Temporal Cortex (LTC) | Transverse temporal cortex, superior temporal gyrus, inferior temporal gyrus, middle temporal gyrus |
| Occipital Cortex (OCC) | Pericalcarine cortex, lingual gyrus, lateral occipital cortex, cuneus cortex |

**Supplementary Table 3.** *Number of fibers removed for each thalamocortical tract during the manual editing step. We conducted minimal manual editing and only removed fibers if they were clearly spurious (e.g., a fiber was "alone" or going in the opposite direction compared to the rest of the fibers).*

| **Thalamocortical Tract** | **N fibers prior to any manual editing** | **N fibers after manual editing** | **Percentage of original tracts maintained after manual editing** |
| --- | --- | --- | --- |
| thalamus-sensorimotor | 6506 | 6488 | 99.7 |
| thalamus-parietal | 9307 | 9273 | 99.6 |
| thalamus-orbitofrontal | 4919 | 4891 | 99.4 |
| thalamus-occipital | 549 | 543 | 98.9 |
| thalamus-medial temporal | 1268 | 1250 | 98.6 |
| thalamus-medial prefrontal | 9274 | 9240 | 99.6 |
| thalamus-lateral temporal | 3006 | 2946 | 98.0 |
| thalamus-lateral prefrontal | 3128 | 3105 | 99.3 |

**Supplementary Table 4.** Group and Age Effects on Thalamocortical Trajectories in Psychosis-spectrum youth vs. Typically Developing Youth vs. Youth with Other Psychopathologies: QSDR and DTI Measures.

| Diffusion Measure | Cortex | Parametric Effects of Group | | | | | | | | | Non-parametric Effects of Age | | | | | | | | | Age Range(s) When Significant Change is Taking Place | | | Age Range(s) When Smoothed Effects of Age Differed between Groups | | |
| --- | --- | --- | --- | --- | --- | --- | --- | --- | --- | --- | --- | --- | --- | --- | --- | --- | --- | --- | --- | --- | --- | --- | --- | --- | --- |
|  |  | TD vs. OP | | | TD vs. PS | | | OP vs. PS | | | TD | | | OP | | | PS | | | TD | OP | PS | TD vs. OP | TD vs. PS | OP vs. PS |
|  |  | T | p | q | T | p | q | T | p | q | F | p | q | F | p | q | F | p | q |  |  |  |  |  |  |
| Quantitative Anisotropy | Lateral Prefrontal | -1.3 | 0.18 | 0.33 | 1.1 | 0.28 | 0.43 | 2.1 | 0.04 | 0.11 | 4.5 | 0.01 | 0.04 | 1.3 | 0.26 | 0.41 | 2.1 | 0.15 | 0.29 | 8.2-13.6, 16.2-23 | ○ | ○ | ○ | ○ | ○ |
|  | Medial Prefrontal | -1.3 | 0.19 | 0.33 | 0.2 | 0.84 | 0.89 | 1.3 | 0.19 | 0.34 | 3.8 | 0.02 | 0.07 | 1.3 | 0.22 | 0.36 | 2.6 | 0.09 | 0.19 | ○ | ○ | ○ | ○ | ○ | ○ |
|  | Orbitofrontal | -1.5 | 0.13 | 0.26 | 0.1 | 0.89 | 0.92 | 1.4 | 0.16 | 0.30 | 4.3 | 0.01 | 0.04 | 1.4 | 0.16 | 0.30 | 2.3 | 0.09 | 0.20 | 8.2-10.9, 15.6-23 | ○ | ○ | ○ | ○ | ○ |
|  | Sensorimotor | -1.4 | 0.15 | 0.29 | -0.4 | 0.70 | 0.78 | 0.9 | 0.36 | 0.51 | 20.5 | 2.2e-16 | 2.1e-15 | 9.7 | 5.2e-05 | 3.4e-04 | 4.8 | 5.4e-03 | 0.02 | 13.4-23 | 13.4-23 | 15.2-23 | ○ | ○ | ○ |
|  | Lateral Temporal | -1.5 | 0.13 | 0.26 | 0.1 | 0.91 | 0.93 | 1.4 | 0.16 | 0.30 | 85.8 | 2.2e-16 | 2.1e-15 | 42.3 | 2.2e-16 | 2.1e-15 | 11.8 | 6.1e-04 | 3.3e-03 | 8.2-23 | 8.2-23 | 8.2-23 | ○ | ○ | ○ |
|  | Medial Temporal | -1.6 | 0.11 | 0.22 | -9.7e-02 | 0.92 | 0.93 | 1.3 | 0.19 | 0.33 | 123.8 | 2.2e-16 | 2.1e-15 | 43.7 | 2.2e-16 | 2.1e-15 | 20.3 | 7.6e-06 | 5.7e-05 | 8.2-23 | 8.2-23 | 8.2-23 | ○ | ○ | ○ |
|  | Parietal | -1.2 | 0.24 | 0.38 | -0.3 | 0.78 | 0.85 | 0.8 | 0.43 | 0.58 | 36.7 | 2.2e-16 | 2.1e-15 | 29.1 | 2.2e-16 | 2.1e-15 | 13.1 | 2.9e-04 | 1.8e-03 | 8.2-23 | 8.2-23 | 12.4-23 | ○ | ○ | ○ |
|  | Occipital | -0.9 | 0.37 | 0.53 | -0.4 | 0.68 | 0.76 | 0.4 | 0.66 | 0.75 | 83.7 | 2.2e-16 | 2.1e-15 | 31.6 | 2.2e-16 | 2.1e-15 | 22.6 | 2.3e-06 | 1.8e-05 | 8.2-23 | 8.2-23 | 8.2-23 | ○ | ○ | ○ |
| Global Fractional Anisotropy | Lateral Prefrontal | -0.4 | 0.66 | 0.75 | -0.4 | 0.67 | 0.76 | 2.7e-02 | 0.98 | 0.98 | 24.5 | 2.2e-16 | 2.1e-15 | 18.0 | 4.2e-07 | 3.7e-06 | 4.3 | 0.04 | 0.11 | 8.2-16.5 | 8.2-15.9 | ○ | ○ | 8.2-10.6, 14.2-17.8 | 8.2-10.6, 14.2-17.9 |
|  | Medial Prefrontal | -1.4 | 0.16 | 0.29 | -3.5 | 4.8e-04 | 2.7e-03 | -1.7 | 0.08 | 0.18 | 23.4 | 2.2e-16 | 2.1e-15 | 15.1 | 1.4e-06 | 1.1e-05 | 3.5 | 0.03 | 0.09 | 8.2-16.7 | 8.2-15.6 | ○ | ○ | ○ | 14.5-16.9 |
|  | Orbitofrontal | -1.6 | 0.10 | 0.21 | -2.0 | 0.05 | 0.13 | -0.2 | 0.81 | 0.87 | 6.3 | 3.8e-03 | 0.02 | 2.4 | 0.11 | 0.22 | 5.1e-02 | 0.83 | 0.88 | 8.2-15.3 | ○ | ○ | ○ | 14.6-17.3 | ○ |
|  | Sensorimotor | -1.9 | 0.06 | 0.14 | -2.9 | 3.5e-03 | 0.01 | -0.8 | 0.42 | 0.57 | 2.4 | 0.06 | 0.15 | 5.6 | 3.3e-03 | 0.01 | 4.1e-02 | 0.85 | 0.89 | ○ | 8.2-12.4, 15.2-23 | ○ | ○ | ○ | 12.2-16.7, 19-23 |
|  | Lateral Temporal | -2.0 | 0.04 | 0.12 | -3.3 | 1.0e-03 | 5.3e-03 | -1.0 | 0.31 | 0.47 | 3.9 | 0.05 | 0.13 | 2.3 | 0.13 | 0.25 | 0.4 | 0.51 | 0.65 | ○ | ○ | ○ | ○ | ○ | ○ |
|  | Medial Temporal | -1.3 | 0.18 | 0.32 | -3.4 | 6.0e-04 | 3.3e-03 | -1.7 | 0.08 | 0.18 | 18.3 | 1.9e-05 | 1.3e-04 | 2.3 | 0.10 | 0.21 | 2.2 | 0.14 | 0.27 | 8.2-23 | ○ | ○ | ○ | ○ | ○ |
|  | Parietal | -2.5 | 0.01 | 0.04 | -4.7 | 3.0e-06 | 2.3e-05 | -1.8 | 0.07 | 0.17 | 3.3 | 0.03 | 0.09 | 1.2 | 0.28 | 0.43 | 1.6 | 0.21 | 0.35 | ○ | ○ | ○ | ○ | ○ | ○ |
|  | Occipital | -1.4 | 0.16 | 0.30 | -3.6 | 2.8e-04 | 1.7e-03 | -1.9 | 0.06 | 0.15 | 2.5 | 0.06 | 0.15 | 3.1 | 0.10 | 0.20 | 1.6 | 0.32 | 0.47 | ○ | ○ | ○ | ○ | ○ | ○ |
| Isotropy | Lateral Prefrontal | -1.4 | 0.15 | 0.29 | 1.2 | 0.22 | 0.36 | 2.3 | 0.02 | 0.08 | 6.1 | 1.6e-03 | 7.6e-03 | 6.3 | 0.01 | 0.04 | 2.3 | 0.10 | 0.21 | 14.1-23 | 8.2-23 | ○ | ○ | ○ | 15.5-17.5 |
|  | Medial Prefrontal | -1.2 | 0.23 | 0.37 | 1.2 | 0.25 | 0.39 | 2.0 | 0.04 | 0.12 | 6.2 | 1.6e-03 | 7.5e-03 | 4.8 | 0.01 | 0.05 | 2.6 | 0.05 | 0.14 | 14.1-23 | ○ | ○ | ○ | ○ | ○ |
|  | Orbitofrontal | -1.3 | 0.20 | 0.34 | 0.8 | 0.44 | 0.59 | 1.7 | 0.08 | 0.18 | 6.7 | 9.8e-04 | 5.1e-03 | 8.1 | 4.4e-03 | 0.02 | 2.8 | 0.04 | 0.12 | 13.8-23 | 8.2-23 | ○ | ○ | ○ | ○ |
|  | Sensorimotor | -0.9 | 0.35 | 0.50 | 0.5 | 0.62 | 0.73 | 1.2 | 0.21 | 0.36 | 27.3 | 2.2e-16 | 2.1e-15 | 27.9 | 3.8e-07 | 3.5e-06 | 11.8 | 4.6e-04 | 2.7e-03 | 11.2-23 | 8.2-23 | 14.1-23 | ○ | ○ | ○ |
|  | Lateral Temporal | -0.8 | 0.45 | 0.59 | 1.4 | 0.15 | 0.29 | 1.9 | 0.06 | 0.15 | 82.8 | 2.2e-16 | 2.1e-15 | 41.5 | 2.2e-16 | 2.1e-15 | 12.0 | 5.4e-04 | 3.0e-03 | 8.2-23 | 8.2-23 | 8.2-23 | ○ | ○ | ○ |
|  | Medial Temporal | -1.0 | 0.32 | 0.47 | 1.5 | 0.14 | 0.27 | 2.1 | 0.03 | 0.10 | 73.3 | 2.2e-16 | 2.1e-15 | 26.6 | 2.2e-16 | 2.1e-15 | 15.3 | 9.5e-05 | 6.0e-04 | 8.2-23 | 8.2-18.6 | 8.2-23 | ○ | ○ | ○ |
|  | Parietal | -0.6 | 0.57 | 0.69 | 0.9 | 0.36 | 0.51 | 1.3 | 0.20 | 0.34 | 106.0 | 2.2e-16 | 2.1e-15 | 55.9 | 2.2e-16 | 2.1e-15 | 27.5 | 4.6e-07 | 4.0e-06 | 8.2-23 | 8.2-23 | 8.2-23 | ○ | ○ | ○ |
|  | Occipital | -0.6 | 0.56 | 0.69 | 0.4 | 0.70 | 0.78 | 0.8 | 0.40 | 0.56 | 107.6 | 2.2e-16 | 2.1e-15 | 32.2 | 2.2e-16 | 2.1e-15 | 33.4 | 2.2e-16 | 2.1e-15 | 8.2-23 | 8.2-20.8 | 8.2-23 | ○ | ○ | ○ |
| Restricted Diffusion Index | Lateral Prefrontal | -1.4 | 0.15 | 0.29 | 1.2 | 0.23 | 0.37 | 2.3 | 0.02 | 0.08 | 5.6 | 2.6e-03 | 0.01 | 5.3 | 0.02 | 0.07 | 2.2 | 0.10 | 0.21 | 14.4-23 | ○ | ○ | ○ | ○ | ○ |
|  | Medial Prefrontal | -1.2 | 0.23 | 0.37 | 1.1 | 0.29 | 0.44 | 1.9 | 0.05 | 0.14 | 5.7 | 2.4e-03 | 0.01 | 3.4 | 0.03 | 0.09 | 2.6 | 0.06 | 0.14 | 14.4-23 | ○ | ○ | ○ | ○ | ○ |
|  | Orbitofrontal | -1.3 | 0.19 | 0.33 | 0.7 | 0.50 | 0.63 | 1.7 | 0.09 | 0.19 | 6.3 | 1.4e-03 | 6.8e-03 | 7.5 | 6.3e-03 | 0.02 | 2.9 | 0.04 | 0.12 | 14.1-23 | 8.2-23 | ○ | ○ | ○ | ○ |
|  | Sensorimotor | -1.0 | 0.31 | 0.47 | 0.4 | 0.69 | 0.77 | 1.2 | 0.23 | 0.37 | 26.4 | 2.2e-16 | 2.1e-15 | 26.7 | 7.0e-07 | 5.8e-06 | 11.0 | 6.7e-04 | 3.5e-03 | 11.8-23 | 8.2-23 | 14.3-23 | ○ | ○ | ○ |
|  | Lateral Temporal | -0.8 | 0.40 | 0.55 | 1.3 | 0.20 | 0.34 | 1.8 | 0.07 | 0.16 | 85.4 | 2.2e-16 | 2.1e-15 | 42.4 | 2.2e-16 | 2.1e-15 | 12.3 | 4.7e-04 | 2.7e-03 | 8.2-23 | 8.2-23 | 8.2-23 | ○ | ○ | ○ |
|  | Medial Temporal | -1.1 | 0.29 | 0.44 | 1.3 | 0.19 | 0.33 | 2.1 | 0.04 | 0.11 | 76.6 | 2.2e-16 | 2.1e-15 | 27.9 | 2.2e-16 | 2.1e-15 | 16.4 | 5.4e-05 | 3.5e-04 | 8.2-23 | 8.2-19 | 8.2-23 | ○ | ○ | ○ |
|  | Parietal | -0.6 | 0.52 | 0.66 | 0.8 | 0.41 | 0.56 | 1.3 | 0.21 | 0.35 | 103.0 | 2.2e-16 | 2.1e-15 | 52.8 | 2.2e-16 | 2.1e-15 | 26.4 | 8.0e-07 | 6.6e-06 | 8.2-23 | 8.2-23 | 8.2-23 | ○ | ○ | ○ |
|  | Occipital | -0.6 | 0.53 | 0.66 | 0.3 | 0.78 | 0.85 | 0.8 | 0.43 | 0.58 | 105.5 | 2.2e-16 | 2.1e-15 | 33.6 | 2.2e-16 | 2.1e-15 | 32.4 | 2.2e-16 | 2.1e-15 | 8.2-23 | 8.2-23 | 8.2-23 | ○ | ○ | ○ |

| Fractional Anisotropy | Lateral Prefrontal | -0.5 | 0.61 | 0.72 | -0.1 | 0.91 | 0.93 | 0.4 | 0.72 | 0.79 | 66.3 | 2.2e-16 | 2.1e-15 | 31.2 | 2.2e-16 | 2.1e-15 | 19.0 | 1.4e-05 | 9.7e-05 | 8.2-17.6 | 8.2-16.8 | 8.2-23 | ○ | 8.2-11.2, 13.9-18.2 | 14.6-17.6 |
| --- | --- | --- | --- | --- | --- | --- | --- | --- | --- | --- | --- | --- | --- | --- | --- | --- | --- | --- | --- | --- | --- | --- | --- | --- | --- |
|  | Medial Prefrontal | -1.1 | 0.27 | 0.42 | -2.4 | 0.02 | 0.06 | -1.1 | 0.28 | 0.43 | 106.3 | 2.2e-16 | 2.1e-15 | 48.7 | 2.2e-16 | 2.1e-15 | 17.2 | 5.1e-07 | 4.4e-06 | 8.2-18 | 8.2-17.6 | 8.2-18.5 | ○ | ○ | ○ |
|  | Orbitofrontal | -1.9 | 0.06 | 0.15 | -1.8 | 0.08 | 0.18 | 0.2 | 0.87 | 0.90 | 28.5 | 2.2e-16 | 2.1e-15 | 7.3 | 3.3e-03 | 0.01 | 4.1 | 0.04 | 0.12 | 8.2-16.7 | 8.2-15.6 | ○ | ○ | 8.2-11, 14.2-18.1 | ○ |
|  | Sensorimotor | -2.3 | 0.02 | 0.07 | -2.2 | 0.03 | 0.08 | 0.1 | 0.91 | 0.93 | 20.5 | 2.2e-16 | 2.1e-15 | 12.6 | 9.7e-06 | 7.2e-05 | 5.5 | 3.5e-03 | 0.01 | 8.2-16.2, 21-23 | 8.2-15.3, 18.8-23 | 8.2-16.9 | ○ | ○ | ○ |
|  | Lateral Temporal | -1.5 | 0.13 | 0.26 | -2.2 | 0.02 | 0.08 | -0.6 | 0.56 | 0.69 | 3.1 | 0.08 | 0.18 | 1.0 | 0.32 | 0.47 | 0.5 | 0.49 | 0.63 | ○ | ○ | ○ | ○ | ○ | ○ |
|  | Medial Temporal | -1.8 | 0.08 | 0.18 | -3.3 | 9.0e-04 | 4.7e-03 | -1.3 | 0.20 | 0.34 | 7.7 | 2.0e-03 | 9.5e-03 | 0.7 | 0.40 | 0.55 | 3.3 | 0.07 | 0.16 | 10.5-23 | ○ | ○ | ○ | ○ | ○ |
|  | Parietal | -2.1 | 0.04 | 0.11 | -3.5 | 4.2e-04 | 2.5e-03 | -1.2 | 0.22 | 0.36 | 30.2 | 2.2e-16 | 2.1e-15 | 24.6 | 2.2e-16 | 2.1e-15 | 4.7 | 0.02 | 0.06 | 8.2-16.5 | 8.2-17.6 | ○ | ○ | ○ | ○ |
|  | Occipital | -0.9 | 0.39 | 0.55 | -3.2 | 1.4e-03 | 6.8e-03 | -2.0 | 0.05 | 0.12 | 20.0 | 2.2e-16 | 2.1e-15 | 20.1 | 2.2e-16 | 2.1e-15 | 2.1 | 0.08 | 0.18 | 8.2-16.3 | 8.2-16.2 | ○ | ○ | ○ | ○ |
| Mean Diffusivity | Lateral Prefrontal | 0.8 | 0.41 | 0.56 | 1.4 | 0.15 | 0.29 | 0.5 | 0.61 | 0.73 | 3.5 | 0.08 | 0.18 | 1.1 | 0.29 | 0.44 | 0.3 | 0.57 | 0.69 | ○ | ○ | ○ | ○ | ○ | ○ |
|  | Medial Prefrontal | 0.5 | 0.65 | 0.74 | 0.4 | 0.69 | 0.77 | -6.0e-02 | 0.95 | 0.96 | 6.4 | 4.2e-03 | 0.02 | 4.9 | 0.03 | 0.08 | 0.2 | 0.68 | 0.76 | 8.2-15.5 | ○ | ○ | 13.9-16.1 | 14.9-17.2 | ○ |
|  | Orbitofrontal | 1.6 | 0.11 | 0.22 | 1.3 | 0.20 | 0.34 | -0.3 | 0.76 | 0.83 | 6.3 | 5.4e-03 | 0.02 | 3.9 | 0.05 | 0.13 | 0.6 | 0.45 | 0.60 | 8.2-15.6 | ○ | ○ | ○ | ○ | ○ |
|  | Sensorimotor | 0.6 | 0.53 | 0.66 | -0.7 | 0.46 | 0.61 | -1.2 | 0.24 | 0.38 | 6.0 | 3.5e-03 | 0.01 | 3.2 | 0.07 | 0.18 | 0.5 | 0.49 | 0.63 | 8.2-14.7, 17.5-23 | ○ | ○ | ○ | 13.7-16.9 | ○ |
|  | Lateral Temporal | -1.0 | 0.32 | 0.47 | -0.6 | 0.53 | 0.67 | 0.3 | 0.73 | 0.80 | 1.0 | 0.44 | 0.59 | 0.9 | 0.36 | 0.51 | 0.3 | 0.56 | 0.69 | ○ | ○ | ○ | ○ | ○ | ○ |
|  | Medial Temporal | 0.7 | 0.50 | 0.64 | 1.3 | 0.18 | 0.33 | 0.5 | 0.59 | 0.71 | 4.0 | 0.05 | 0.12 | 0.3 | 0.61 | 0.73 | 4.7 | 0.03 | 0.09 | ○ | ○ | ○ | ○ | ○ | ○ |
|  | Parietal | 1.4 | 0.17 | 0.32 | -4.9e-02 | 0.96 | 0.97 | -1.2 | 0.22 | 0.36 | 12.4 | 1.3e-05 | 9.6e-05 | 6.5 | 9.8e-03 | 0.04 | 0.3 | 0.59 | 0.71 | 8.2-15.9 | 8.2-16.1 | ○ | ○ | ○ | ○ |
|  | Occipital | 0.5 | 0.65 | 0.74 | 1.0 | 0.30 | 0.45 | 0.5 | 0.63 | 0.74 | 4.6 | 0.03 | 0.09 | 5.8 | 4.3e-03 | 0.02 | 0.2 | 0.67 | 0.76 | ○ | 8.2-14.4, 17-23 | ○ | 13-15.8, 20.2-23 | ○ | 13.9-17.6 |
| Radial Diffusivity | Lateral Prefrontal | 0.9 | 0.39 | 0.55 | 1.3 | 0.19 | 0.33 | 0.4 | 0.71 | 0.78 | 8.1 | 1.3e-03 | 6.7e-03 | 3.5 | 0.08 | 0.18 | 1.2 | 0.27 | 0.42 | 8.2-15.9 | ○ | ○ | ○ | ○ | ○ |
|  | Medial Prefrontal | 0.7 | 0.49 | 0.63 | 0.9 | 0.36 | 0.51 | 0.2 | 0.86 | 0.90 | 11.4 | 4.0e-05 | 2.7e-04 | 6.5 | 9.8e-03 | 0.04 | 0.7 | 0.40 | 0.55 | 8.2-15.9 | 8.2-16 | ○ | ○ | 14.5-17.6 | ○ |
|  | Orbitofrontal | 1.8 | 0.07 | 0.16 | 1.6 | 0.10 | 0.21 | -0.2 | 0.82 | 0.87 | 8.6 | 4.9e-04 | 2.7e-03 | 3.3 | 0.08 | 0.18 | 0.5 | 0.46 | 0.61 | 8.2-15.6 | ○ | ○ | ○ | 14.5-17.3 | ○ |
|  | Lateral Temporal | -0.5 | 0.62 | 0.73 | 0.2 | 0.86 | 0.90 | 0.6 | 0.56 | 0.69 | 1.0 | 0.34 | 0.50 | 0.2 | 0.64 | 0.74 | 0.6 | 0.43 | 0.58 | ○ | ○ | ○ | ○ | ○ | ○ |
|  | Medial Temporal | 0.9 | 0.38 | 0.53 | 1.8 | 0.07 | 0.16 | 0.8 | 0.43 | 0.58 | 7.1 | 7.7e-03 | 0.03 | 0.7 | 0.39 | 0.55 | 5.9 | 0.02 | 0.05 | 8.2-23 | ○ | ○ | ○ | ○ | ○ |
|  | Sensorimotor | 1.1 | 0.27 | 0.43 | -0.1 | 0.91 | 0.93 | -1.1 | 0.29 | 0.44 | 5.8 | 3.4e-03 | 0.01 | 3.7 | 0.04 | 0.10 | 0.2 | 0.62 | 0.73 | 8.2-14.4, 16.7-23 | ○ | ○ | ○ | 13.4-16.9 | 14.2-16.7 |
|  | Parietal | 1.8 | 0.08 | 0.18 | 1.1 | 0.28 | 0.43 | -0.6 | 0.54 | 0.68 | 11.0 | 3.6e-05 | 2.5e-04 | 6.1 | 0.01 | 0.04 | 0.3 | 0.64 | 0.74 | 8.2-15.5, 18.4-23 | 8.2-15.7 | ○ | ○ | ○ | ○ |
|  | Occipital | 0.6 | 0.55 | 0.69 | 1.8 | 0.08 | 0.18 | 1.0 | 0.33 | 0.48 | 3.6 | 0.06 | 0.14 | 6.5 | 2.2e-03 | 0.01 | 0.3 | 0.57 | 0.70 | ○ | 8.2-14.5, 17.1-23 | ○ | ○ | ○ | 8.2-10.4, 13.9-17.8 |
| Axial Diffusivity | Lateral Prefrontal | 0.6 | 0.55 | 0.68 | 1.5 | 0.14 | 0.27 | 0.7 | 0.47 | 0.61 | 0.9 | 0.33 | 0.48 | 1.7 | 0.20 | 0.34 | 0.3 | 0.60 | 0.72 | ○ | ○ | ○ | ○ | ○ | ○ |
|  | Medial Prefrontal | -0.1 | 0.89 | 0.92 | -0.7 | 0.49 | 0.63 | -0.5 | 0.64 | 0.74 | 0.2 | 0.79 | 0.85 | 0.3 | 0.62 | 0.73 | 0.2 | 0.64 | 0.74 | ○ | ○ | ○ | ○ | ○ | ○ |
|  | Orbitofrontal | 0.7 | 0.47 | 0.62 | 0.3 | 0.77 | 0.83 | -0.4 | 0.71 | 0.78 | 1.8 | 0.18 | 0.32 | 2.8 | 0.10 | 0.20 | 0.4 | 0.53 | 0.66 | ○ | ○ | ○ | ○ | ○ | ○ |
|  | Lateral Temporal | -1.9 | 0.06 | 0.15 | -2.1 | 0.03 | 0.09 | -0.2 | 0.86 | 0.90 | 1.9 | 0.17 | 0.31 | 2.9 | 0.09 | 0.19 | 1.3e-02 | 0.91 | 0.93 | ○ | ○ | ○ | ○ | ○ | ○ |
|  | Medial Temporal | 0.2 | 0.84 | 0.89 | 0.2 | 0.85 | 0.89 | -1.5e-02 | 0.99 | 0.99 | 0.2 | 0.64 | 0.74 | 5.5e-02 | 0.82 | 0.87 | 2.3 | 0.13 | 0.26 | ○ | ○ | ○ | ○ | ○ | ○ |
|  | Sensorimotor | -0.4 | 0.67 | 0.76 | -1.9 | 0.06 | 0.15 | -1.2 | 0.22 | 0.36 | 5.8 | 7.4e-03 | 0.03 | 5.6 | 0.02 | 0.06 | 1.0 | 0.33 | 0.48 | 8.2-15.4 | ○ | ○ | 13.7-15.8 | ○ | ○ |
|  | Parietal | 0.2 | 0.83 | 0.88 | -2.5 | 0.01 | 0.05 | -2.3 | 0.02 | 0.07 | 13.6 | 3.6e-05 | 2.5e-04 | 6.2 | 0.01 | 0.05 | 1.7 | 0.19 | 0.33 | 8.2-17.2 | 8.2-23 | ○ | ○ | ○ | ○ |
|  | Occipital | 0.2 | 0.88 | 0.91 | -0.4 | 0.69 | 0.77 | -0.5 | 0.63 | 0.74 | 10.1 | 1.5e-03 | 7.4e-03 | 3.3 | 0.05 | 0.12 | 1.7e-02 | 0.91 | 0.93 | 8.2-23 | ○ | ○ | 11.9-16.1, 18.7-23 | ○ | 14.3-16.9 |

**Supplementary Table 5.** DTI effects of sex for thalamocortical tracts. Bold text denotes significant effects.

| Diffusion  Measure | Brain  Region | t value | Cohen’s d | p value | **q value** | Direction of Effect |
| --- | --- | --- | --- | --- | --- | --- |
| Fractional Anisotropy | Lateral Prefrontal | 3.1 | -0.2 | 0.002 | 0.006 | M>F |
|  | Medial Prefrontal | 4.6 | -0.3 | 4.16e-06 | 8.88e-05 | M>F |
|  | Orbitofrontal | 3.8 | -0.2 | 1.62e-04 | 8.66e-04 | M>F |
|  | Sensorimotor | 3.1 | -0.2 | 0.002 | 0.005 | M>F |
|  | Lateral Temporal | 4.1 | -0.2 | 5.04e-05 | 5.02e-04 | M>F |
|  | Medial Temporal | 1.9 | -0.1 | 0.055 | 0.076 | • |
|  | Parietal | 3.4 | -0.2 | 7.81e-04 | 0.003 | M>F |
|  | Occipital | 0.7 | -0.0 | 0.491 | 0.533 | • |
| Mean Diffusivity | Lateral Prefrontal | -2.2 | 0.1 | 0.028 | 0.048 | F>M |
|  | Medial Prefrontal | -2.3 | 0.1 | 0.023 | 0.040 | F>M |
|  | Orbitofrontal | -3.3 | 0.2 | 9.87e-04 | 0.003 | F>M |
|  | Sensorimotor | -1.0 | 0.1 | 0.295 | 0.337 | • |
|  | Lateral Temporal | -0.7 | 0.0 | 0.461 | 0.509 | • |
|  | Medial Temporal | -1.9 | 0.1 | 0.056 | 0.076 | • |
|  | Parietal | -3.1 | 0.2 | 0.002 | 0.005 | F>M |
|  | Occipital | -0.4 | 0.0 | 0.723 | 0.758 | • |
| **Radial Diffusivity** | Lateral Prefrontal | -2.1 | 0.1 | 0.035 | 0.053 | • |
|  | Medial Prefrontal | -2.6 | 0.1 | 0.011 | 0.022 | F>M |
|  | Orbitofrontal | -3.4 | 0.2 | 7.94e-04 | 0.003 | F>M |
|  | Lateral Temporal | -1.4 | 0.1 | 0.148 | 0.182 | • |
|  | Medial Temporal | -1.8 | 0.1 | 0.073 | 0.095 | • |
|  | Sensorimotor | -1.5 | 0.1 | 0.128 | 0.161 | • |
|  | Parietal | -3.4 | 0.2 | 5.86e-04 | 0.003 | F>M |
|  | Occipital | -0.5 | 0.0 | 0.590 | 0.629 | • |
| **Axial Diffusivity** | Lateral Prefrontal | -2.0 | 0.1 | 0.041 | 0.060 | • |
|  | Medial Prefrontal | -1.5 | 0.1 | 0.123 | 0.158 | • |
|  | Orbitofrontal | -2.5 | 0.1 | 0.013 | 0.024 | F>M |
|  | Lateral Temporal | 0.8 | -0.0 | 0.435 | 0.489 | • |
|  | Medial Temporal | -2.0 | 0.1 | 0.042 | 0.060 | • |
|  | Sensorimotor | 0.1 | -3.93e-03 | 0.944 | 0.959 | • |
|  | Parietal | -1.8 | 0.1 | 0.066 | 0.088 | • |
|  | Occipital | 0.0 | -1.81e-03 | 0.974 | 0.974 | • |

**Supplementary Table 6.** QSDR effects of sex for thalamocortical tracts. Bold text denotes significant effects.

| Diffusion Measure | Brain  Region | t value | Cohen’s d | p value | q value | Direction of Effect |
| --- | --- | --- | --- | --- | --- | --- |
| Quantitative Anisotropy | Lateral Prefrontal | -3.4 | 0.2 | 7.41e-04 | 0.003 | F>M |
|  | Medial Prefrontal | -4.4 | 0.3 | 1.05e-05 | 1.69e-04 | F>M |
|  | Orbitofrontal | -2.5 | 0.1 | 0.012 | 0.022 | F>M |
|  | Sensorimotor | -4.1 | 0.2 | 4.94e-05 | 5.02e-04 | F>M |
|  | Lateral Temporal | -1.1 | 0.1 | 0.275 | 0.320 | • |
|  | Medial Temporal | -2.7 | 0.2 | 0.007 | 0.016 | F>M |
|  | Parietal | -2.1 | 0.1 | 0.032 | 0.051 | • |
|  | Occipital | 2.1 | -0.1 | 0.036 | 0.054 | • |
| Global Fractional Anisotropy | Lateral Prefrontal | 1.4 | -0.1 | 0.172 | 0.207 | • |
|  | Medial Prefrontal | 3.4 | -0.2 | 8.28e-04 | 0.003 | M>F |
|  | Orbitofrontal | 1.1 | -0.1 | 0.251 | 0.297 | • |
|  | Sensorimotor | 2.7 | -0.2 | 0.007 | 0.016 | M>F |
|  | Lateral Temporal | 3.4 | -0.2 | 8.01e-04 | 0.003 | M>F |
|  | Medial Temporal | -0.2 | 0.0 | 0.809 | 0.835 | • |
|  | Parietal | 4.0 | -0.2 | 7.16e-05 | 5.21e-04 | M>F |
|  | Occipital | 2.7 | -0.2 | 0.007 | 0.016 | M>F |
| Isotropy | Lateral Prefrontal | -4.0 | 0.2 | 5.49e-05 | 5.02e-04 | F>M |
|  | Medial Prefrontal | -4.8 | 0.3 | 1.71e-06 | 6.10e-05 | F>M |
|  | Orbitofrontal | -3.5 | 0.2 | 5.07e-04 | 0.002 | F>M |
|  | Sensorimotor | -3.8 | 0.2 | 1.61e-04 | 8.66e-04 | F>M |
|  | Lateral Temporal | -2.5 | 0.1 | 0.012 | 0.022 | F>M |
|  | Medial Temporal | -3.2 | 0.2 | 0.001 | 0.004 | F>M |
|  | Parietal | -2.1 | 0.1 | 0.033 | 0.051 | • |
|  | Occipital | 2.6 | -0.1 | 0.009 | 0.020 | M>F |
| Restricted Diffusion Index | Lateral Prefrontal | -4.0 | 0.2 | 7.32e-05 | 5.21e-04 | F>M |
|  | Medial Prefrontal | -4.8 | 0.3 | 1.91e-06 | 6.10e-05 | F>M |
|  | Orbitofrontal | -3.4 | 0.2 | 7.70e-04 | 0.003 | F>M |
|  | Sensorimotor | -3.9 | 0.2 | 1.03e-04 | 6.57e-04 | F>M |
|  | Lateral Temporal | -2.4 | 0.1 | 0.016 | 0.028 | F>M |
|  | Medial Temporal | -3.1 | 0.2 | 0.002 | 0.005 | F>M |
|  | Parietal | -2.2 | 0.1 | 0.030 | 0.049 | F>M |
|  | Occipital | 2.6 | -0.1 | 0.011 | 0.022 | M>F |

**Supplementary Figure 1A-D.** White matter tracts between the thalamus and A. lateral prefrontal cortex, B. medial prefrontal cortex, C. orbitofrontal cortex, D. sensorimotor cortex.

**Supplementary Figure 1F-J.** White matter tracts between the thalamus and F. lateral temporal cortex, G. left medial temporal cortex, H. right medial temporal cortex, I. parietal cortex, and J. occipital cortex.****

**Supplementary Figure 2.** Group differences in thalamocortical global fractional anisotropy in Typically Developing Youth (blue) vs. Youth with Other Psychopathologies (yellow) vs. Psychosis-spectrum youth (red). **I.-P.** Group Differences in thalamocortical fractional anisotropy in Typically Developing Youth (blue) vs. Youth with other Psychopathologies (yellow) vs. Psychosis-spectrum youth (red). The y-axis represents residualized data (age and sex regressed out) from the thalamocortical tracts.

**Supplementary Figure 3.** Neurodevelopmental trajectories of radial diffusivity in thalamocortical tracts of psychosis-spectrum youth, typically developing youth, and youth with other psychopathologies. Partial residual plots of radial diffusivity trajectories in tracts between the thalamus and A. lateral prefrontal cortex, B. medial prefrontal cortex, C. orbitofrontal cortex, D. sensorimotor cortex, E. lateral temporal cortex, F. medial temporal cortex, G. parietal cortex, and H. occipital cortex. The partial residual plots reflect the relationship between age and radial diffusivity, given the covariates in the model. For each group, the thick line reflects the line of best fit. The bars underneath the age plots reflect the derivative of the slope, i.e., the rate of change taking place at a particular age. Darker blue indicates that there is a stronger decrease in radial diffusivity taking place at that age, while brighter red indicates a stronger increase in radial diffusivity. Dotted lines and areas of lighted shaded blue indicate times when there was a significant “difference in smooths” between Typically Developing and Psychosis Spectrum youth.


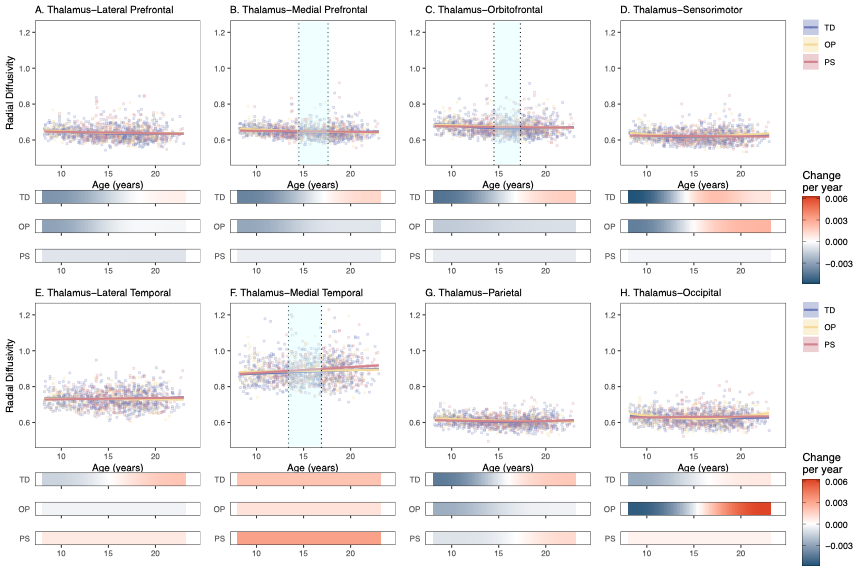


**Supplementary Figure 4**Neurodevelopmental trajectories of mean diffusivity in thalamocortical tracts of psychosis-spectrum youth, typically developing youth, and youth with other psychopathologies. Partial residual plots of mean diffusivity trajectories in tracts between the thalamus and A. lateral prefrontal cortex, B. medial prefrontal cortex, C. orbitofrontal cortex, D. sensorimotor cortex, E. lateral temporal cortex, F. medial temporal cortex, G. parietal cortex, and H. occipital cortex. The partial residual plots reflect the relationship between age and mean diffusivity, given the covariates in the model. For each group, the thick line reflects the line of best fit. The bars underneath the age plots reflect the derivative of the slope, i.e., the rate of change taking place at a particular age. Darker blue indicates that there is a stronger decrease in mean diffusivity taking place at that age, while brighter red indicates a stronger increase in mean diffusivity. Dotted lines and areas of lighted shaded blue indicate times when there was a significant “difference in smooths” between Typically Developing and Psychosis Spectrum youth.


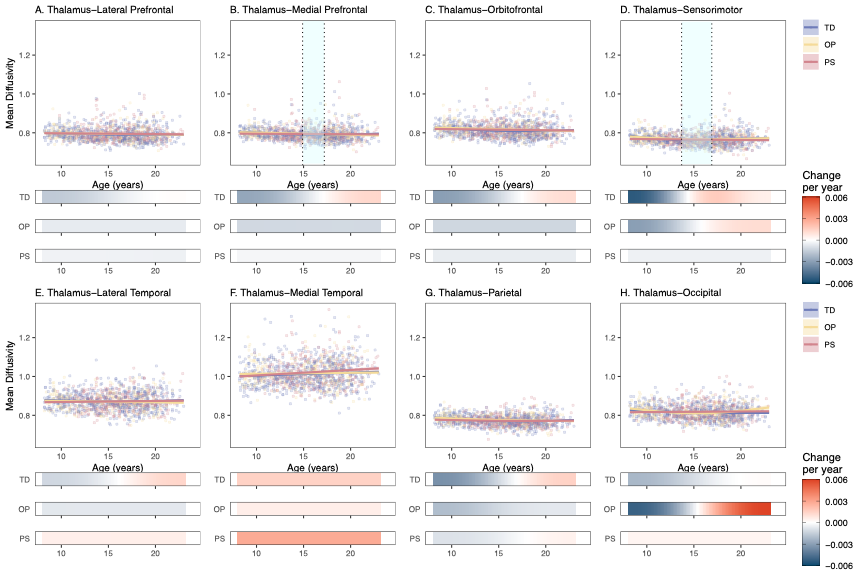

Supplement: Supplementary file 1 — Supplemental Material [file 41537_2023_411_MOESM1_ESM.docx]
